# Penetration of SDF and AgF from the infected dentine towards the unaffected tooth structure

**DOI:** 10.3389/froh.2023.1298211

**Published:** 2023-12-11

**Authors:** Riaan Mulder, Nicoline Potgieter, Naeemah Noordien

**Affiliations:** ^1^ Department of Restorative Dentistry, Faculty of Dentistry, University of the Western Cape, Bellville, South Africa; ^2^ Department of Orthodontics & Paediatric Dentistry, Faculty of Dentistry, University of the Western Cape, Bellville, South Africa

**Keywords:** ion movement, silver diamine fluoride, water based silver fluoride, infected dentine, affected dentine, special care dentistry

## Abstract

**Background:**

The use of SEM-EDS line scan analysis to evaluate the movement of ions from dental materials towards the tooth structure and the concept of ion movement is well established. This analysis technique was used to determine the ion movement of two commercially available silver- and fluoride-containing products.

**Methods:**

This study aimed to compare the elemental analysis of primary molar teeth treated with silver diamine fluoride (SDF) and water-based silver fluoride (AgF) and to analyse the penetration of SDF and AgF from the infected dentine towards the healthy dentine. The teeth were cleaned from debris and contaminants off the roots and stored until use. A total of 15 primary molars with large active cavitated lesions, not extending into the pulp (specimens), were divided into three test groups: silver diamine fluoride (SDF) (*n* = 5), water-based silver fluoride (AgF) (*n* = 5), and deionised water (W) (*n* = 5) as the control group. The teeth were sectioned, embedded, and received SEM-EDS line scans. The line scan had a total length of 82.65 μm. The visible end of the infected dentine and the start of the more affected dentine were chosen as the starting point to ensure that the infected caries' line distribution towards the affected dentine's transition area was as standardized as possible. Therefore, the infected dentine length of the scan was 22.80 μm (8 scan points of 2.85 μm apart), and the affected dentine, including the healthy dentine, was 59.8 μm (21 scan points). The SEM-EDS line scan from each specimen determined the average fluoride, iodide, and silver weight percentage for that specimen.

**Results:**

The 15 sample SEM-EDS line scans were used to determine the average ion movement in wt%. The Kruskall–Wallis test and Tukey's HSD test were completed at a *p* < 0.05. SDF and AgF presented no significant fluoride movement in terms of the weight percentage. There was, however, significantly more fluoride movement from infected caries to the healthy dentine with SDF and AgF (*p* = 0.0010053) compared to the control specimens treated with deionised water. There was no significant difference between SDF and AgF for the movement of the iodide (*p* = 0.5953) and silver (*p* = 0.3708) from infected caries to the healthy dentine.

**Conclusion:**

SDF and AgF easily penetrated through infected caries and affected tooth structure to the healthy dentine for the line scan of 82.65 μm. There was no significant difference between SDF and AgF for the movement of ions within the infected dentine nor in the affected/healthy dentine.

## 1. Introduction

The use of scanning electron microscopy—energy dispersive spectroscopy (SEM-EDS) analysis to evaluate the movement of ions from the glass ionomer restorative cement into the tooth structure and the concept of ion movement is well documented in the literature. These ions and their release into the tooth structure through ion exchange have been established as the mechanism that enables glass ionomer cement to remineralise the demineralised dentine (1). During subsequent research, Gjorgievska et al. (2) indicated that aluminium, fluoride, magnesium, silicon, and strontium were incorporated into the dentine from Fuji IX (GC Corporation, Japan). Although the distance of ion movement was not assessed using SEM-EDS line scans as in this present *in vitro* study, Gjorgievska et al. (2) could conclude that the variation of the ion movement between different teeth would be limited by the dense crystalline nature of the tooth structure (2). This *in vitro* study, therefore, assessed the movement of ions from silver diamine fluoride (SDF) and water-based silver fluoride (AgF) into the healthy dentine through the infected and affected dentine. SDF is known to easily penetrate through 1–1.5 mm dentine discs where the surface was treated with 17% EDTA to open the dentinal tubules (3). Therefore, the depth of penetration and the corresponding weight percentage change will be insightful to the remineralising properties of the SDF and AgF materials. The use of SEM-EDS can be considered to be destructive as the teeth need to be sectioned and prepared. The use of Micro-CT evaluation has shown value in assessing microstructures such as interglobular dentine (4, 5) and is a method that should be considered for incorporation in future research to augment the caries remineralisation assessment of affected and infected dentine, preceding SEM-EDS analysis. Assessing if the AgF where the ammonium was removed as per the previous generation known as SDF becomes clinically relevant to clinicians using AgF in communities where refrigeration is no longer a requirement for AgF.

The hypothesis of this study was that the weight percentage of ion movement from the SDF and AgF towards the healthy dentine structure through the infected and affected dentine would be no different.

## 2. Materials and methods

This study aimed to compare the ion movement from the infected dentine towards the healthy dentine through elemental analysis of primary molar teeth treated with silver diamine fluoride (SDF) and water-based silver fluoride (AgF).

### 2.1. Sample size calculation

Based on the fluoride values obtained from Knight et al. (6), the fluoride ion mean of the control group was 4 and that of the test group was 10, with a standard deviation of 3. Assuming a pooled standard deviation of 3 units, the study would require a sample size of 4 teeth per group. The sample size of 5 is, therefore, more than sufficient to achieve a power of 80% and a level of significance of 5% (two-sided) for detecting a true difference in means between the test and the reference groups.

### 2.2. Specimen collection and SDF/AgF treatment

All teeth used in this study were collected from a pool of teeth that were extracted as part of a comprehensive treatment plan based on the patient's needs and not for the purpose of this study. All patients or parents/legal guardians signed written consent for the treatment plans independently of this research and gave consent for teeth to be used for anonymised research purposes. The teeth used are not traceable to the patients. Specimens were stored appropriately and disposed of according to medical waste guidelines, once testing took place. The study was approved by the University of the Western Cape Research Ethical Committee with approval number: BM22/7/3.

The teeth were cleaned from debris and contaminants from the roots and stored under refrigeration in 1% thymol distilled water until use. When the experiment commenced, teeth were rinsed with double distilled water. A total of 15 primary molars with large active cavitated lesions, not extending into the pulp (specimens), were divided into three test groups: silver diamine fluoride (SDF) (*n* = 5), water-based silver fluoride (AgF) (*n* = 5), and deionised water (W) (*n* = 5) as the control group.

SDF (Riva Star, SDI Limited, Australia) and AgF (Riva Star Aqua, SDI Limited, Australia) were the commercially available products and they were used as per the manufacturer's instructions. The control group was represented with deionised water. The treated teeth were stored in 80% humidity at 37°C for 2 weeks before SEM-EDS assessment. This method allowed for the removal of the confounding factor of excessive moisture that could carry ions deeper than the natural remineralisation progression of the ions into the tooth structure.

### 2.3. SEM-EDS analysis of the sectioned teeth

**Figure 1:**
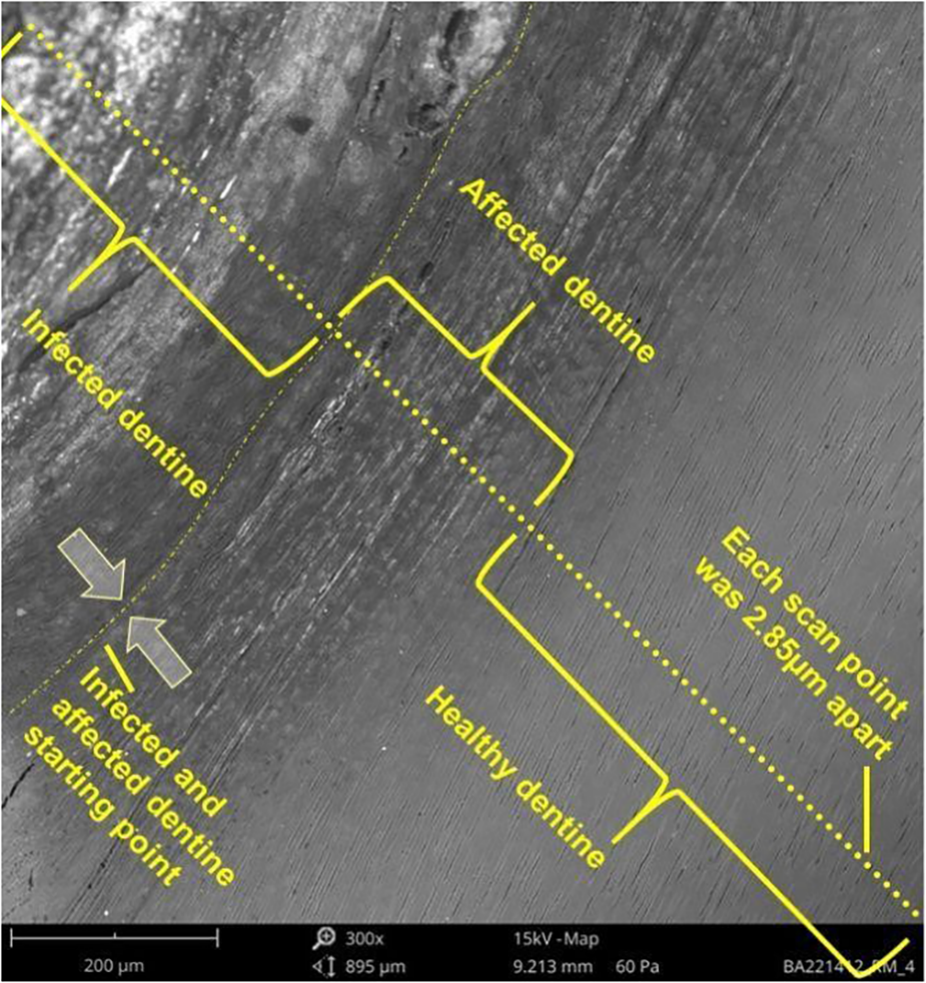
Illustration of the sectioned tooth as seen under the SEM.

The teeth that were sectioned and embedded in clear acrylic resin received an SEM-EDS line scan from the infected tooth structure toward the healthy dentine structure to assess the ion elemental transition in weight percentage concentration (wt%). This SEM-EDS line scan was repeated five times per sample and the average of the corresponding μm positions was used to calculate the average elemental wt% within groups. Each SEM-EDS line scan was 50 μm apart from the adjacent scanning lines. All five specimens from each group were critical-point dried in a desiccator, invested in clear acrylic, polished, and sputter-coated with carbon prior to SEM-EDS analysis. The SEM-EDS line scan from each specimen determined the average fluoride, iodide, and silver weight percentage for that specimen at each point on the line scan. The scanning electron microscopy unit (Hitachi S-4800 FEG Scanning Electron Microscope, Hitachi Ltd, Tokyo, Japan) was used to examine the surface morphology of the specimens to perform the line scan. The line scan had a total length of 82.65 μm. The visible end of the infected dentine to the affected dentine was chosen as the starting point to ensure that the infected caries' line distribution towards the affected dentine's transition area was as standardized as possible. Therefore, the infected dentine length of the scan was 22.80 μm (8 scan points of 2.85 μm apart) and that of the affected dentine, including the healthy dentine, was 59.8 μm (21 scan points) (Figure 1). Images were obtained at FOV: 944 µm, Mode: 15 kV—Map, Detector: BSD Full. The measurements of the scan points were 2.85 μm apart and wt% percentage concentration for the ion elements present.

### 2.4. Baseline elements

The baseline fluoride, silver, and iodide weight percentage concentration (wt%) in the healthy dentine was assessed with SEM-EDS 4 mm away from the infected tooth structure for the teeth used in this *in vitro* investigation and was chosen to be 1.4% per weight. The fluoride results obtained from the line scan did not receive any data correction and were used as analysed per line scan point. There was no iodide nor silver present in these sections of the teeth.

## 3. Results

**Figure 2:**
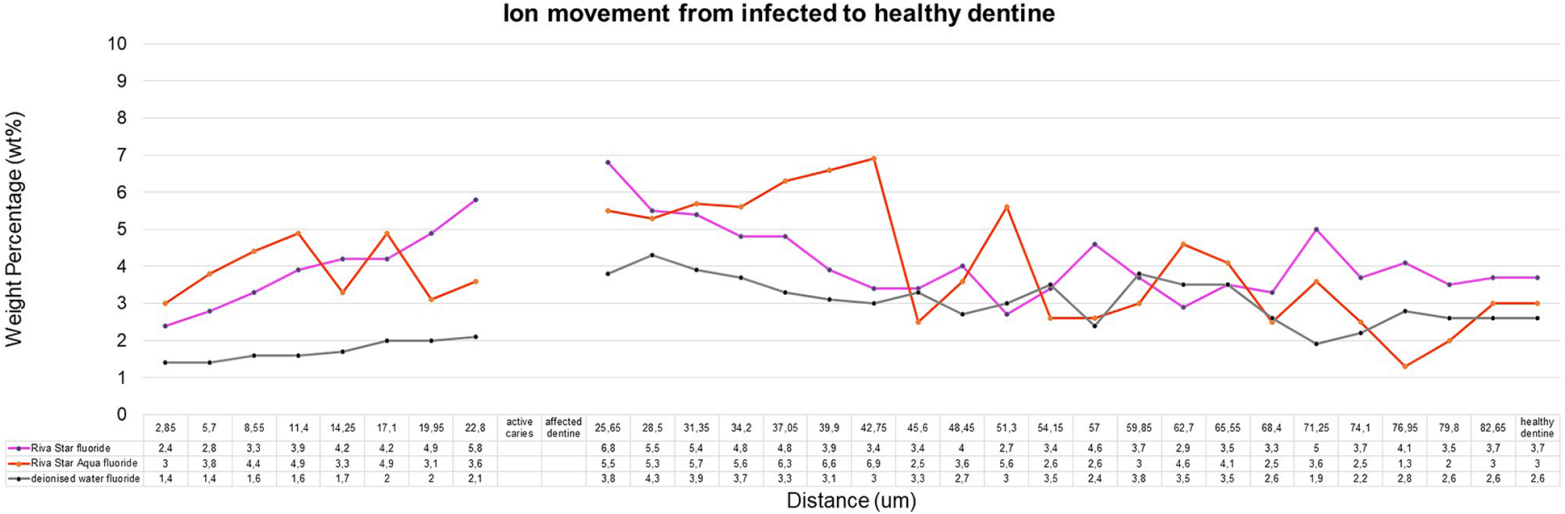
Fluoride ion movement in wt%.

**Figure 3:**
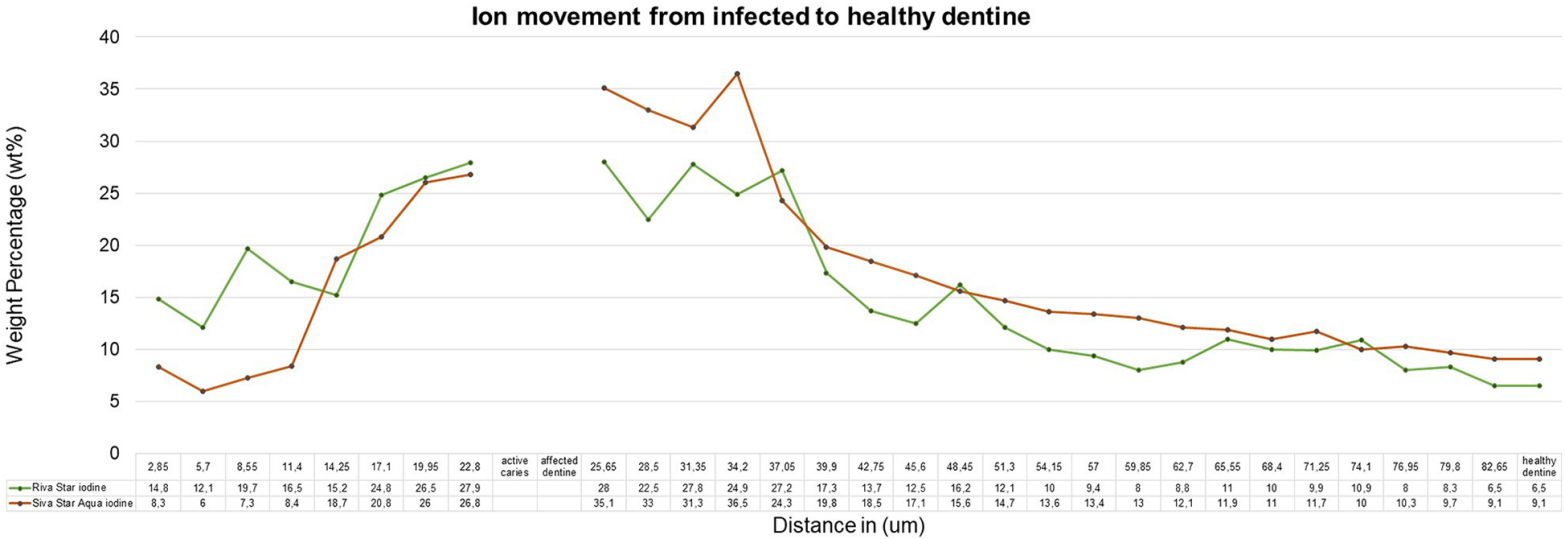
Iodine ion movement in wt%.

**Figure 4:**
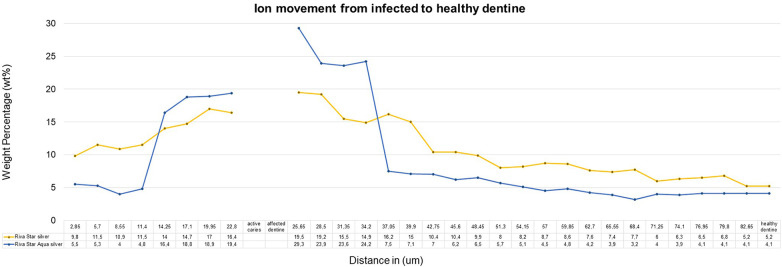
Silver ion movement in wt%.

The sample (*n* = 5) SEM-EDS line scans were used to determine the average ion movement in wt%. The Kruskall–Wallis test determined there was a difference among the groups. Tukey's HSD test determined where the significant difference was between groups at a *p* < 0.05. SDF and AgF presented significantly more fluoride movement from infected caries to the healthy dentine, with SDF and AgF showing significantly more (*p* = 0.0010053) movement compared to the control specimen treated with deionised water (Figure 2). There was no significant difference between SDF (Riva Star) and AgF (Riva Star Aqua) for the movement of the fluoride (*p* = 0.8999), iodide (*p* = 0.5953) (Figure 3), and silver (*p* = 0.3708) (Figure 4) from infected caries to the healthy dentine.

**Figure 5:**
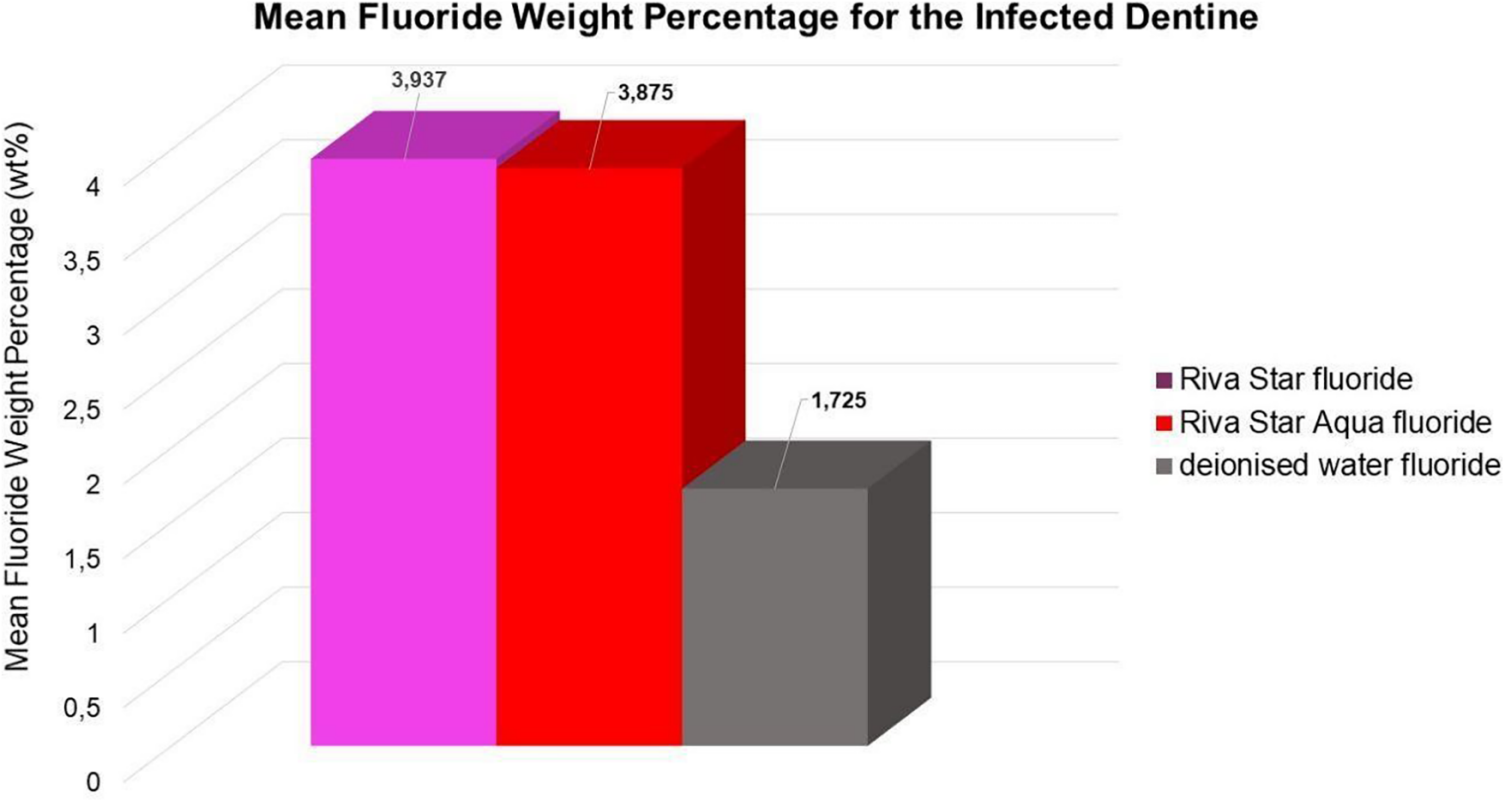
Mean fluoride weight percentage in the infected dentine.

There were no significances found between AgF (Riva Star Aqua) and SDF (Riva Star) with regard to the wt% present in the affected and healthy dentine cumulatively for fluoride wt% (Figure 5), silver wt%, and iodide (*p* < 0.05).

## 4. Discussion

The hypothesis that the ion movement from the AgF (Riva Star Aqua) and SDF (Riva Star) towards the healthy dentine structure through the infected and affected dentine would be no different was accepted.

When a carious tooth is restored with a glass ionomer cement it is well established that the carious teeth develop an inter-diffusion zone with a width of 1–2 µm (7). The ion movement from Fuji IX (GC Corporation, Japan) and Riva Self Cure (SDI Limited, Australia) glass ionomer restorative cement was also assessed by Knight et al. (6). Their investigation illustrated the movement of ions into the tooth up to 75 µm for Fuji IX and Riva Self Cure, but their graphs illustrated a sharp decline of ion wt% after 20 µm (6).

The use of 38% SDF encourages the remineralisation of carious lesions when looking at depth, mineral density, and remineralisation (8). SDF, when exposed to demineralised dentine, increases the mineral organic content and increases the size of the hydroxyapatite crystals in the treated dentine (9). This contributes to the improvement of the mechanical properties of the remineralised dentine thus preventing further demineralisation. This was also demonstrated by Iijima and Onuma (10), who reported that the addition of fluoride into the lattice of remnant crystals changed and decreased the solubility of the apatite. This is further supported by Zhi et al. (11), who found that topical application of silver and fluoride ions increases the mineral density of demineralised dentine lesions. This *in vitro* study confirmed this conclusion and was able to quantify the weight percentage for the various ions. The use of a remineralisation product, especially on special care patients, that is nearly odourless, has no tissue irritation, and has a physiological pH like Riva Star Aqua could be considered favourable properties. The mineralisation and arrestation of dental caries is the primary function of SDF and AgF products and this role must be optimal. This *in vitro* analysis compared ion movement in weight percentage concentration (wt%) of Riva Star (SDF) to establish if the physical material properties would hinder the mineralisation effect of the Riva Star Aqua (AgF).

It would be expected that the composition of SDF (Riva Star) and AgF (Riva Star Aqua) would play a role in the ion wt% potential of the material that is available to move through the infected and affected dentine into the healthy tooth structure. Although there were no significant differences (*p* > 0.05) between the wt% concentrations of SDF (Riva Star) and AgF (Riva Star Aqua) in the infected tooth structure, affected dentine, healthy dentine nor in the line scan as a whole, there were interesting wt% concentrations that correlate well with the formulation of the Riva Star and Riva Star Aqua product content. The safety data sheets presented by Riva Star and Riva Star Aqua have similar weight concentration percentages of fluoride (5%) and silver (25%) (12). The SEM-EDS detection of calcium-fluoride mineral content is lower for infected dentine and increase progressively towards the unaffected and healthy dentine. It has been documented that Riva Star forms a calcium fluoride precipitation on the surface of the carious lesion as protein-based globules (13), so the higher fluoride wt% of Riva Star Aqua on the surface (Figure 2) due to the neutral pH could be investigated further with confocal microscopy to visualize what exact mechanism results in the higher fluoride wt%. It seemed that the wt% for Riva Star Aqua started lower than Riva Star in the infected tooth structure but the line scan wt% increased through the affected tooth structure towards the affected dentine for iodide (Figure 3) and silver (Figure 4), with Riva Star still achieving the highest wt% of fluoride at the start of the affected dentine compared to Riva Star Aqua. The use of ammonia in Riva Star is postulated, based on microstructural tooth research of ammonia adsorption (14), to result in a greater collagen expansion and binding compared to water alone (15). Adding nitric acid assists with the collagen expansion and stabilisation of the components to achieve a stable shelf life without refrigeration. The storage advantage of Riva Star Aqua (AgF) not needing to be refrigerated like other SDF products is ideal for clinical and outreach events within urban and rural settings. This ability to be used in rural areas where electricity or continuous refrigeration is limited speaks to the United Nations' sustainable development goals of good health and well-being as communities, which otherwise would not have been able to receive this new formulation of AgF, can be reached. Besides speaking to the UN's goal, it also highlights the efforts of manufacturers in creating and delivering innovation to the curative and preventative dentistry sphere. The literature from a 6-month follow-up clinical study (16) supported the findings that Riva Star Aqua and Riva Star are effective in arresting the advancement of caries. Riva Star Aqua is an ideal addition to the armamentarium of the clinician for wider population caries treatment methodologies. Additionally, teeth treated with Riva Star Aqua have shown on a hydroxyapatite microcellular surface an antibiofilm effect on the microbial biofilm (17).

The penetration of the two products through the infected and affected dentine towards the healthy dentine through the affected caries is also an important consideration. The cumulative mean fluoride weight percentage for Riva Star and Riva Star Aqua was similar (Figure 5). As the line scan analysis of the wt% continued towards the arrested caries area, it was noted that the point scan of 25.65 µm into the infected dentine seemed to be the “tipping point” for where the mineral structure became denser towards the affected dentine. The affected dentine also had the initial part closest to the infected dentine where cariogenic bacteria penetrated and was also mineralised by the various SDF products. The interdiffusion zone of Riva Star Aqua has been shown to be an effective product against *S. mutans* when assessed with other SDF products (18). The Riva Star Aqua had a non-significant higher wt% for silver (Figure 4) and iodide (Figure 3) in the various aspects of the line scan compared to Riva Star, which had a slightly higher fluoride wt% (Figure 2). This could be explained by the aqua component of Riva Star Aqua, which drove the wt% through the infected dentine into the affected tooth structure, whereas the evaporation of the solvent could have possibly delayed the initial penetration of the ions through infected caries for the Riva Star, explaining the much higher wt% of iodide (14.83 wt%) and silver (9.8 wt%) at the first line scan point in the infected dentine. Whereas Riva Star Aqua had 8.3 wt% and 5.5 wt% of iodide and silver, respectively, and could penetrate deeper due to the aqueous content and potential lack of solvent evaporation upon application.

From the literature and reports using various techniques, the silver concentration of Riva Star Aqua was estimated to be 266,477 ppm (26.65% of the content) and the fluoride concentration was estimated to be 42,429 ppm (18) compared to the fluoride concentration of Riva Star at 3,922 ppm (3). The different techniques provide different ppm values for fluoride, and further research should be completed to have the available fluoride values determined with the same methodologies, as it was out of the scope of this research. The Safety Data Sheet documents of the products indicated the same fluoride wt% for Riva Star and Riva Star Aqua. The ion movement in the various parts of the teeth was also found to be similar (Figures 2, 5). Riva Star and Riva Star Aqua at contact with the infected dentine indicated the silver wt% in the infected dentine was 9.8 wt% and 5.5 wt%, respectively, indicating that the silver phosphate layer (19) formed by Riva Star also served as a reservoir and allowed deeper penetration of the silver and iodine. This also explains the lower fluoride wt% close to the caries surface of Riva Star-treated teeth, as the first container of Riva Star contains the fluoride where the initial deeper penetration was supported by the second bottle in the system. The line scan progression towards the affected dentine indicated an increase in the wt% and at the last infected dentine reading Riva Star illustrated a 16.4 wt% and Riva Star Aqua showed 19.4 wt%. The first line scan point in the affected dentine showed the following: Riva Star 19.5 wt% and Riva Star Aqua 29.3 wt%. Riva Star Aqua would, therefore, also offer resistance to future cariogenic attacks, since silver has been cited to react and bind to hydroxyapatite (20). As the line scan progressed from the affected dentine to the healthy dentine, Riva Star (5.2 wt%) had a consistently higher silver concentration than Riva Star Aqua (4.1 wt%).

For both silver and iodide, the 34.2–37.05 µm measurement from the infected dentine presented Riva Star Aqua with a higher wt% at 34.2 µm with 36.5 wt% iodide and 24.2 wt% silver compared to Riva Star with 24.9 wt% iodide (Figure 3) and 14.9 wt% silver (Figure 4). The crossover of the iodide concentration was at multiple line scan points between Riva Star and Riva Star Aqua.

When Riva Star and Riva Star Aqua came into contact with the infected dentine of the tooth samples, the iodide wt% in the infected dentine was 27.9 wt% and 26.6 wt%, respectively. The line scan progression towards the affected dentine indicated an increase in the wt% and at the last infected dentine reading the Riva Star illustrated a 26 wt% and the Riva Star Aqua registered a much higher reading at 35.1 wt%. The first line scan point in the affected dentine was Riva Star (22.5 wt%), with Riva Star Aqua having once again a much higher wt% (33 wt%). As the line scan progressed from the affected dentine to the healthy dentine, Riva Star (8.5 wt%) had a consistently lower iodide concentration than Riva Star Aqua (9.1 wt%) (Figure 3).

After 14 days of the infected dentine lesion, the control group presented with 1.4 wt% fluoride and this increased to 2.1 wt% at the perceived end of the infected dentine. From the affected dentine, 3.8 wt% fluoride was present and stabilized at 2.6 wt%. Riva Star (2.4 wt%) and Riva Star Aqua (2.0 wt%) were similar at the start of the line scan of the infected dentine of the tooth structure. Both Riva Star and Riva Star Aqua presented a significantly higher fluoride concentration in the infected dentine lesion compared to the control. This was to be expected as caries would reduce the wt% of the fluoride and other ions usually present in dentine. At the perceived border of the infected dentine and the transition into affected dentine, the wt% in the infected dentine for Riva Star was 5.8 wt% and 3.6 wt% for Riva Star Aqua. The start of the affected dentine showed an increase: 6.8 wt% for Riva Star and 5.5 wt% for Riva Star Aqua. At both interfaces the wt% between the two products were not statistically significant. Both Riva Star and Riva Star Aqua illustrated a fluoride wt% penetration for the entire length of the line scan to the 82.65 µm healthy dentine at 3.7 wt% and 3 wt%, respectively. Therefore, the combination of ion movement well through the infected dentine towards the healthy dentine from Riva Star and Riva Star Aqua provides the building blocks for maintaining remineralisation of the affected dentine collagen (20) (Figure 2).

## 5. Conclusion

There was no significant difference between SDF (Riva Star) and AgF (Riva Star Aqua) for the movement through the AgF and SDF groups of infected caries nor the wt% in the affected/healthy dentine. Limitations to this study are the degree of demineralisation of the infected and affected dentine between teeth, this is also why the calcium wt% was not discussed. The SDF and AgF in the *in vitro* setting had no artificial saliva exposure, so the calcium wt% changes that would take place in such a scenario were not a confounding factor. This cannot be standardised, but the wt% of fluoride in the teeth could be measured in order to determine the ion movement. Additionally, the visibly infected caries towards the affected caries area were determined to be the transition point, as seen by the line scan wt% values. The use of micro-CT evaluation of the carious lesion before and after treatment of the tooth is a valuable methodology to add to future research.

## Data Availability

The raw data supporting the conclusions of this article will be made available by the authors, without undue reservation.
